# Predicting acute stress recovery: A resilience index of physiological responses to Trier Social Stress Test

**DOI:** 10.1016/j.ijchp.2025.100652

**Published:** 2025-12-18

**Authors:** Li Liang, Chris Xie Chen, Ngan Yin Chan, Suk-Yu Yau, Yan Liu, Shirley Xin Li, Yun Kwok Wing, Tatia Mei-Chun Lee, Wai Kai Hou

**Affiliations:** a Laboratory of Neuropsychology and Human Neuroscience, The University of Hong Kong, Hong Kong SAR, China; b InnoCentre of Clinical Neuropsychology, The University of Hong Kong, Hong Kong SAR, China; c Centre for Psychosocial Health, The Education University of Hong Kong, Hong Kong SAR, China; d Li Chiu Kong Family Sleep Assessment Unit, Department of Psychiatry, Faculty of Medicine, The Chinese University of Hong Kong, Hong Kong SAR, China; e Department of Rehabilitation Sciences, The Hong Kong Polytechnic University, Hong Kong SAR, China; f Mental Health Research Center, The Hong Kong Polytechnic University, Hong Kong SAR, China; g Department of Computing, The Hong Kong Polytechnic University, Hong Kong SAR, China; h Sleep Research Clinic and Laboratory, Department of Psychology, The University of Hong Kong, Hong Kong SAR, China; i Li Ka Shing Institute of Health Sciences, Faculty of Medicine, The Chinese University of Hong Kong, Hong Kong SAR, China; j Guangdong-Hong Kong Joint Laboratory for Psychiatric Disorders, China; k Department of Psychology, The Education University of Hong Kong, Hong Kong SAR, China

**Keywords:** Stress, Psychological resilience, Positive affect, Mental health, Trier Social Stress Test

## Abstract

Previous theoretical and empirical research has highlighted the predictive utility of different physiological reactivity and recovery patterns during acute stress for long-term mental health outcomes. Timely identification of mental health risk can be achieved by integrating these multiple temporal responses to characterize adaptive, dynamic resilience factors and then generating a resilience index. This study aimed to generate a resilience index to characterize the adaptive and dynamic resilient physiological responses and identify the predictors of these responses from a wide array of candidate predictors of psychological resilience in previous studies. Trier Social Stress Test (TSST) was used to induce acute stress responses in a sample of 248 participants (56.0 % female). Principal component analyses (PCA) were employed to integrate cortisol and cardiovascular responses to the TSST. The resilience index, comprising of the PCA reactivity and recovery scores, was related to better mental health. Using the least absolute shrinkage and selection operator regression, 25 of the 48 predictors were identified as critical ones, including baseline physiological activity, coping and emotion regulation strategies (e.g., positive reappraisal and instrumental support seeking), positive affective style and emotional reactivity, cognitive functions (e.g., interference inhibition), and demographic factors (e.g., minor medical conditions) (absolute magnitude of coefficients=0.402–3.865). These findings highlighted the importance of considering stress reactivity and recovery and physiological stress responses to understand the resilience factors, offering significant insight into developing wearable cognitive behavioral adjustment protocols to promote recovery from stress and hence mental well-being.

## Introduction

Psychological resilience refers to the maintenance of positive mental health or quick recovery from significant psychiatric symptoms during and after an adverse life event or a period of difficult life circumstances (Bonanno, 2004; Kalisch et al., 2017). Resilience is observed in both major life adversities and minor daily hassles. The adaptive and dynamic responses to daily stressors could reflect resilience as an inherent capacity for change, calling for more research on whether and how resilience factors embedded in multiple domains (e.g., affective, biological) align with each other in daily life (Kalisch et al., 2015; Ong & Leger, 2022; Troy et al., 2023).

There is also growing consensus towards the multilevel and multidimensional nature of resilience factors to adversity (Infurna & Luthar, 2017; Troy et al., 2023). The hypothalamic–pituitary–adrenal (HPA) axis and sympathetic–adrenal–medullary (SAM) axis are two primary biological systems in response to a stressor. Physiological stress responses consist of two phases, reactivity and recovery. Reactivity is considered as an adaptive response to stress (McEwen, 1998), but heightened reactivity could be a risk factor for health outcomes (McEwen, 1998; Turner et al., 2020). Prolonged reactivity reflects one of the patterns of allostatic load that reflects impaired recovery, denoting the wear and tear of lasting exposure to heightened bodily responses resulting from repeated or chronic environmental challenges that an individual perceives stressful (McEwen, 1998). Relatively low reactivity coupled with fast recovery, hereafter referred to as resilient responses, could alleviate the allostatic load due to frequent encounters with stressful events and be beneficial to the sustainment of mental health in the face of daily hassles (Agorastos & Chrousos, 2022; Feder et al., 2019). This conceptualization highlights the importance of considering both stress reactivity and recovery in understanding the resilience factors. In contrast, individuals with lower reactivity and delayed recovery, or those with higher reactivity but faster recovery, are more likely to maintain psychological functioning compared to those with high reactivity and delayed recovery.

Cumulative evidence showed that heightened cardiovascular and cortisol reactivity to acute psychological stress was positively associated with psychiatric symptoms. For example, the positive association between cortisol reactivity to Trier Social Stress Test (TSST) and depressive symptoms has been observed among both young and older healthy adults (Puig-Perez et al., 2016; Strickland et al., 2024). In addition, higher levels of cortisol responses to TSST during pregnancy predicted higher levels of postpartum depressive symptoms whether in the presence or absence of a large-scale stressful context (Beech et al., 2023; Nierop et al., 2006). By utilizing a trauma film paradigm, Schultebraucks et al. (2019) found that heightened physiological response to TSST was prospectively associated with more intrusive memories related to analogue traumatic experiences in the first few days after the experiment. Other studies also demonstrated that cortisol and cardiovascular reactivity to psychological stress were positively associated with heightened state anxiety and PTSD symptoms (Engel et al., 2023; Grace et al., 2022; Lee et al., 2022). Among young adults exposed to a conflictual interpersonal experimental condition, the association between childhood maltreatment and internalizing symptoms was significant at high but not low levels of increase in cortisol (Hagan et al., 2014). Within a parent–adolescent interaction paradigm, the positive associations between stressful family life events and adolescents’ internalizing symptoms and externalizing behaviors were significant only at higher levels of cortisol reactivity (Steeger et al., 2017). Some previous studies, nonetheless, showed a positive association between blunted stress reactivity and mental health conditions (e.g., Carroll et al., 2007; Ginty et al., 2021), albeit without contextualizing the association in stressful conditions, suggesting that both high and low reactivity could be detrimental to mental health such as depression, anxiety, and post-traumatic stress disorder (Carroll et al., 2009; Turner et al., 2020).

Compared with a large body of studies on the associations between acute stress reactivity and mental health, the link between acute stress recovery and mental health is relatively understudied, despite the fact that recovery to baseline functioning is the sequential process following reactivity to an acute stressor (Agorastos & Chrousos, 2022; McEwen, 1998). Meta-analytic evidence is available to show that higher cortisol concentration during recovery period was found on individuals with depression relative to non-depressed individuals (Burke et al, 2005). Among adolescents of lower magnitude of cortisol recovery following stress induction, the associations between their depressive symptoms and lifetime stressful events were stronger than those of greater cortisol recovery (Quinn et al., 2018). Among individuals without a psychiatric diagnosis, steeper rates of reactivity and recovery were associated with higher levels of depressive symptoms whereas lower rates with elevated anxiety symptoms (Fiksdal et al., 2019). What is less known in the current evidence base is how an integrated response could be identified from multiple physiological systems of stress responses instead of considering each interrelated response (e.g., heart rate, blood pressure, cortisol) separately.

More investigation is also needed on the risk and protective factors pertinent to the associations between resilient physiological stress responses and the maintenance of mental health following adversity. A recent affect-regulation framework of psychological resilience has suggested that families of affect-regulation strategies predict more adaptive short-term consequences (e.g., affective experience, physiological responses, social processes) of stressful encounters, which, in turn, shape psychological resilience in the contexts of the characteristics of adversity and the broader socioecological context (Troy et al., 2023). Another conceptual framework for the neurobiological study of resilience proposed that the relationships between risk and protective factors and resilient physiological responses can offer insights into the specific mechanisms of resilience through which various predictors (e.g., demographic factors, risk and protective factors, socioecological variables) operate (Kalisch et al., 2015). Positive psychological states or traits have been found to be associated with lower levels of HPA reactivity while hostility and aggression were associated with higher cardiovascular reactivity (Chida & Hamer, 2008). Cortisol and/or cardiovascular recovery to acute stress was slower on individuals reporting higher levels of general life stress, negative affect, and rumination (Chida & Hamer, 2008; Radstaak et al., 2011; Stewart et al., 2013). More research is needed to investigate the associations between resilient physiological responses and different predictors of psychological resilience reported in previous studies, such as cognitive and behavioral coping, positive and negative states and traits, stressor exposure, and demographic characteristics (Bonanno et al., 2007; Schäfer et al., 2022).

### The present study

This study aims to construct a resilience index and identify its predictors from a wide array of candidate predictors of psychological resilience in previous studies (Bonanno et al., 2007; Schäfer et al., 2022). Physiological responses to acute stress characterized by low reactivity and fast recovery were considered as reflecting adaptive responses to daily stressors (Agorastos & Chrousos, 2022; Feder et al., 2019). A machine learning approach was used to develop and finetune the prediction model for the resilient responses with a comprehensive array of psychological and behavioral predictors. This approach enabled the selection of a smaller subset of predictors that are most important in predicting resilient acute stress responses and therefore improved the accuracy and interpretability of the resulting model. To avoid the potential confounding effects of clinically significant mental health conditions (Allen et al., 2014), we studied the acute stress responses on a sample of healthy individuals free of any lifetime and current psychiatric diagnoses.

## Methods

### Participants and procedure

Ethics approval was obtained from the Institutional Review Board of the University of Hong Kong. Between October 2020-March 2023, 248 participants were recruited via advertisement, printed and social media, and from the FAMILY Cohort (Leung et al., 2017), which comprises a participant registry of ∼30,000 local residents. Inclusion criteria were age 18–45 years, secondary school or above education level, and Chinese fluency. Exclusion criteria included (1) current or past major physical or neurological conditions; (2) current or past psychiatric diagnoses such as affective and anxiety disorders, schizophrenia and addiction; (3) any medication or other treatment received within two weeks before the study that might affect the endocrinological system; and (4) (for females) pregnancy or breastfeeding. Structured Clinical Interview for DSM-5 Disorders, Clinician Version (SCID-CV) by experienced interviewers was utilized to screen past or current clinically significant psychiatric conditions.

Eligible participants gave their written informed consent and were invited to join the experiment in the afternoon. Before the experiment, participants were instructed to (1) avoid consuming food or beverage within the last hour; (2) avoid strenuous exercise or teeth-brushing within 2 h, (3) abstain from consuming caffeine and tobacco on the day of the experiment; and (4) abstain from consuming alcohol within 24 h of the experiment. Upon arrival at the laboratory, each participant provided the first saliva sample. They then took a 30 min rest in a quiet room, where they read materials containing emotionally neutral content. This allowed the cardiovascular functioning and cortisol level to approach a resting baseline. Following the resting phase, participants’ second saliva sample, baseline blood pressure (BP), and heart rate (HR) measurements were collected. Subsequently, the participants were taken to the experimental room to undergo the TSST. BP and HR were assessed three times at each phase of TSST. Upon completion of the TSST, the participant was taken back to the quiet room and provided the third saliva sample. The fourth to seventh saliva samples were collected with a 20 min interval. BP and HR during the recovery phase were recorded at 35 min after the TSST. Psychometric measures unrelated to stress and distress such as trait affect and trait resilience were administered after the collection of the fifth saliva sample. Other measures closely relevant to stress and distress such as psychiatric distress and stressor exposure were collected after the final saliva sample.

### Experiment paradigm

Acute stress responses of participants were elicited using the TSST paradigm, a standardized experiment protocol widely adopted in laboratory settings (Allen et al., 2014; Kirschbaum et al., 1993). The TSST comprised three successive stages, each lasting for five minutes: (1) anticipation/preparation, (2) speech, and (3) mental arithmetic. To begin, participants were asked to imagine they were attending an interview for their "ideal job," during which they must deliver a five-minute impromptu speech to a panel of expert examiners. The anticipatory/preparatory phase started immediately after the instruction, during which participants prepared for the speech alone. In the subsequent speech phase, participants delivered their speech to a panel of three judges, one male and two females, all dressed in white laboratory gowns. The judges remained emotionless and speechless unless the participant was unable to speak for the full five minutes, at which point the "chief" judge might ask prompt questions. Dummy camera and audio devices were installed in the room to enhance the realism of the job interview experience. After the speech phase, participants were asked to perform a mental arithmetic task requesting them to subtract 17 from 2023, speaking out the answer loudly after each calculation and starting over if they made a mistake.

### Physiological measures

Salivary cortisol. Salivary cortisol samples were collected using the Salivette Cortisol Kit (Sarstedt, cat. no. 51.1534.500). For each sample collection, the participant was instructed to chew a cotton swab for 60 s to fully soak the swab with saliva. The swab was then placed into the Salivette tube, and saliva samples were retrieved from the tube by centrifugation at 3000 g for 5 min. Cortisol concentration was determined with liquid chromatography-tandem mass spectrometry analysis (LC-MS/MS), which is considered a highly accurate and sensitive method of analyzing salivary cortisol (van den Ouweland et al., 2012). The sample collected immediately before the participant received instructions for the TSST was considered as the baseline level (T1). Subsequent samples collected after the TSST were labeled sequentially from T2 to T6. Peak was defined as the highest cortisol value among T1-T6 (Khoury et al., 2015). Cortisol reactivity was computed as the area under the curve with respect to increase (AUCi; Khoury et al., 2015). Cortisol concentration of the last sample (minimum value at group level) denoted cortisol recovery (Miller et al., 2018).

Heart rate (HR) and Blood pressure (BP). HR and BP were measured at the third minute of each phase using QardioArm, a wireless blood pressure monitor. To begin, the participants sat in an upright position with their back supported and feet flat on the ground. The cuff was placed around their upper left arm with its bottom edge approximately 2–3 cm above the bend of the participants’ elbow. The pulse rate was recorded as an interchangeable measure of HR. The peak HR was determined as the highest value recorded during one of the four 5-minute measurements of both the baseline and the TSST. HR reactivity was indicated by the AUCi of its trajectory. The HR of the recovery stage indicated HR recovery. The diastolic blood pressure (DBP) and systolic blood pressure (SBP) were recorded for calculating mean arterial pressure (MAP) using the formula [DBP + (SBP – DBP)/3]. The highest MAP recorded during one of the three 5-minute measurements of the TSST was deemed the peak MAP. Consistent with the calculation of cortisol and HR reactivity and recovery, the AUCi of the MAP trajectory and the MAP at the recovery stage were considered as BP reactivity and recovery, respectively.

### Questionnaires

Psychometric measures. Exposures to daily stressors and major life events were measured with the Daily Hassle Scale (Holm & Holroyd, 1992) and the Life Stress Index (Nan et al., 2012; Weathers et al., 2013), respectively. Psychological distress was assessed using the 28-item General Health Questionnaire (Goldberg & Hillier, 1979) (Cronbach’s alpha = 0.89). The Profile of Mood States (Grove & Prapavessis, 1992) was used to assess positive and negative emotion states at baseline, after TSST, and during recovery (Cronbach’s alphas = 0.89–0.96). Perceived stress was assessed with the perceived stress scale (Cohen et al., 1983) (Cronbach’s alpha = 0.86). Coping and emotion regulation strategies were assessed using the Cognitive Emotion Regulation Questionnaire short form (Garnefski & Kraaij, 2006) and Brief-COPE (Carver et al. 1997) (Cronbach’s alphas = 0.58–0.95). Affective styles were measured using Chinese Affect Scale (Hamid & Cheng, 1996) (Cronbach’s alphas = 0.89–0.91) and Emotion Reactivity Scale (Nock et al., 2008) (Cronbach’s alpha = 0.95). Trait resilience was measured using the Connor-Davidson Resilience Scale (CD-RISC) (Connor & Davidson, 2003) (Cronbach’s alpha = 0.91). Self-efficacy was assessed with the New General Self-Efficacy scale (Chen et al., 2001) (Cronbach’s alpha = 0.92). The six-item UCLA-Loneliness Scale (Neto et al., 2014) was used to measure perceived loneliness (Cronbach’s alpha = 0.87). Immediate memory/auditory attention, working memory, cognitive flexibility, inattentiveness, and cognitive inhibition were assessed through a battery of neuropsychological tests. Detailed information regarding the measurements of the predictors is documented in the Supplementary Material.

Demographics. Information about participants’ gender, age in years, marital status, education level, employment status, monthly household income, and medical history were also collected.

### Data analysis

Descriptive statistics (mean, standard deviation [SD], etc.) were obtained to describe the sample characteristics of the three physiological responses for all timepoints. Missing data related to physiological measures (< 1 %) was handled using K-nearest neighbor (KNN) imputation with *caret* package in R (Kuhn, 2008).

The generalized additive models (GAMs) were constructed using the MGCV package (Wood, 2017) in R to examine the experimental manipulation of the TSST and estimate the trajectories of physiological responses for each individual. The GAM is a flexible statistical framework for modelling complex linear and non-linear fixed and random effects (Hastie & Tibshirani, 1986) and thus is appropriate for modeling non-linear temporal changes in physiological stress responses during the experiment in the current study. A gamma distribution with a log link function was applied for both cortisol and cardiovascular responses (i.e., BP and HR). The area under the curve with respect to increase (AUCi) was calculated to indicate reactivity levels while accounting for the time factor. The curves of physiological responses were estimated by the GAMs with participants and slopes as random effects. Fixed effect of time was set by using Gaussian process spline as the smooth function. The factor smooth function was used to estimate the random effects of time and participants.

To differentiate between cortisol non-responders and responders, we defined cortisol non-responders as those exhibiting a peak reactivity of <15.5 % of their baseline level (Miller et al., 2013). This criterion allowed us to identify distinct cortisol response trajectories for the two groups of participants (Figure S1). The cortisol levels of the non-responders peaked at the arrival sampling and were significantly higher than those of the cortisol responders, subsequently following a decreasing trend over time. In contrast, the cortisol levels of the responders decreased from arrival to baseline, then increased after the TSST, peaking 20 min post-TSST before returning to baseline. Given these differences, cortisol trajectories were modelled using two GAMs separately, one for responders and one for non-responders. In contrast, blood pressure and heart rate responses were comparable between groups (Figures S2 and S3), therefore analyses for these were conducted on the full sample.

Using the *psych* package (Revelle, 2024) in R, two separate principal component analyses (PCAs) were conducted on the values of cortisol, BP, and HR at reactivity and recovery phases, respectively. Before doing the PCA, descriptive statistics (e.g., histogram, skewness) of the raw values and the Shapiro-Wilk test were used to checked the normality of the data distribution. Skewed data (skewness > 3) and non-normally distributed data (Shapiro-Wilk *p* < .05) were pre-processed using either log transformation or inverse hyperbolic sine transformation to achieve normality or approximate normality. Eigenvalue (> 1.0) was used to determine the number of component(s). The component scores of the positive associations with reactivity and recovery values of the three physiological measures denoted the reactivity and recovery scores, respectively. Specifically, given that the recovery period was consistent for all participants, lower recovery scores indicated greater return to baseline functioning across individuals, and thus suggested faster recovery. Because low reactivity (i.e., lower reactivity score) and fast recovery (i.e., lower recovery score) were hypothesized as adaptive stress responses (Agorastos & Chrousos, 2022; Feder et al., 2019), as shown in Eq. 1, the square root of the product of the reactivity score and recovery score was used to indicate resilience (i.e., resilience score). The resilience index was multiplied by –1 to align with the intuitive understanding that a higher score indicates greater levels of resilience.

(1) $$\text{Resilience}\;\text{index}=-\sqrt{\text{PCA}\;\text{Reactivity}\;\times \;\text{PCA}\;\text{Recovery}}$$

The validity of this index was tested by examining its main effect and the interaction effect between daily stressors and resilience index on psychological distress, controlling for the effects of major life events and demographics in a multiple linear regression (Masten et al., 2021). To avoid multicollinearity, raw scores of daily stressors and resilience index were centered among sample means. If an inverse association between the resilience index and psychological distress is observed, it may suggest a protective (compensatory) effect of resilient physiological stress responses on mental health. In addition, if an interaction effect is significant, indicating a weaker association between daily stressors and psychological distress is observed among high-resilience individuals, it may also suggest a protective (buffering) effect of the index, whereby these individuals adapt better to daily stress and experience less psychological distress despite encountering stressful events. The multiple regression model was performed using *jamovi* version 2.4.11 (The jamovi project, 2023).

### Prediction model

A total of 48 predictors were derived from baseline scores of physiological functioning, the aforementioned psychometric measurements, and demographic information. Missing data of the predictor values (< 1 %) was handled using K-nearest neighbor (KNN) imputation. The least absolute shrinkage and selection operator (LASSO) regression model was employed to identify the most relevant variables for predicting the resilience index by imposing a penalty on the magnitude of the regression coefficients and effectively shrinking some coefficients to zero. It performed both variable selection and regularization in a unified process, which improved the generalizability and interpretability of the resulting model (Tibshirani, 1996). LASSO regression model has been increasingly used to identify predictors of trajectories of resilience and psychopathology in recent research (Chen et al., 2022; Liu et al., 2023). The *caret* (Kuhn, 2008) and *glmnet* (Friedman et al., 2010) packages were used to construct the LASSO regression in the R platform.

To enhance model performance, random resampling with replacement was employed as a data augmentation approach on the original participants, generating a synthetic sample of 500 for subsequent predictive modelling. A 10-fold cross-validation with 50 repetitions procedure was adopted to select the optimal shrinkage parameter (i.e., lambda) from 2000 predefined lambda values for the LASSO model. For each lambda value, the sample (*N* = 500) was randomly partitioned into 10 folds, the model was then computed and trained on all but one of the folds, with the remaining fold serving as a test fold once. These procedures were repeated 50 times. The average model performance was calculated for the 500 different held-out sets. The lambda value with the lowest average root-mean-squared-error (RMSE) was selected to obtain the coefficients of the predictors. These coefficients were rescaled from 0 to 100 for denoting variable importance, with the predictor of the greatest magnitude assigned a score of 100. The final LASSO regression model was refitted on the original participants to obtain the *R^2^* and to calculate the RMSE in order to examine any significant discrepancies between the original and augmented samples.

## Results

### Trajectories of acute stress responses

**Fig. 1:**
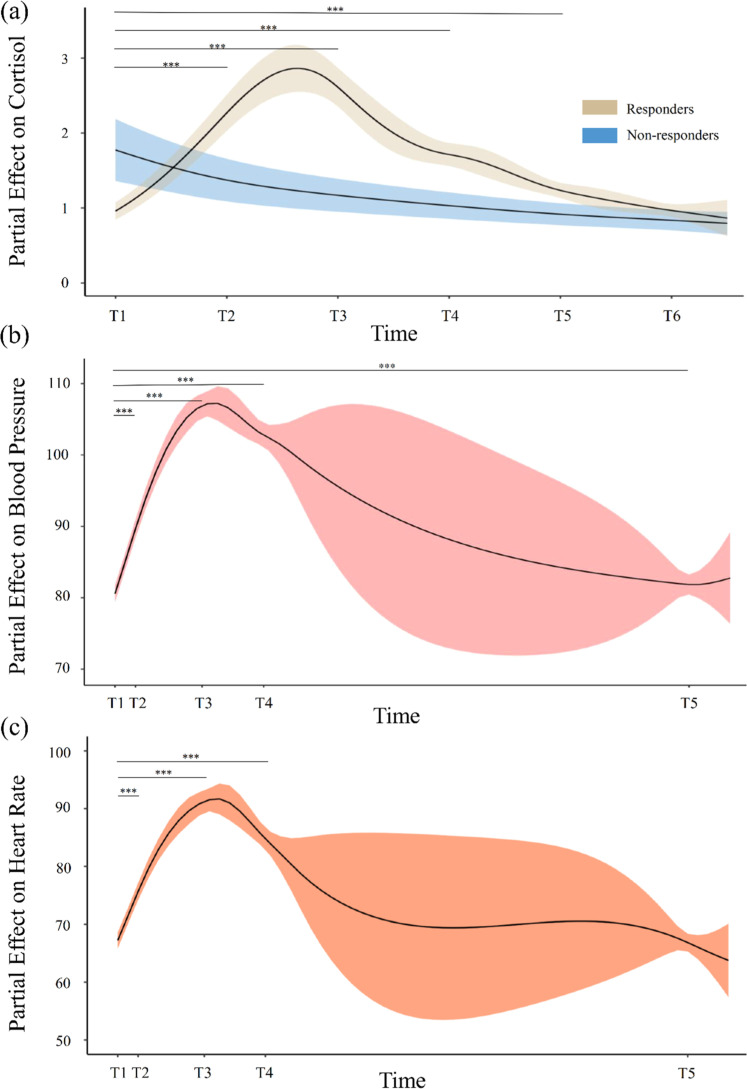
Smooth term of physiological activities across the experiment. The solid line represents the fixed partial effect of time on (a) cortisol concentration (b) blood pressure and (c) heart rate. The shaded area represents the upper and lower bounds of an approximated 95 % confidence interval. For (a), T1=Baseline, T2=Post TSST, T3=20mins Post TSST, T4=40mins Post TSST, T5=60mins Post TSST, T6=80mins Post TSST; Group comparisons were conducted on responders across time. For (b) and (c), T1=Baseline, T2=TSST−Preparation, T3=TSST−Speech, T4=TSST−Calculation, T5=Recovery. *** *p* < .001 (Hommel−corrected).

Table 1 summarizes the demographic characteristics of the sample and Table 2 shows the descriptive statistics of cortisol, BP, and HR. Time was a significant non-linear predictor of cortisol, BP, and HR (Table 3), showing a non-linear pattern of change over the course of the experiment. For cortisol responders, cortisol concentration peaked approximately 10 min after TSST (*p* < .001), declined to baseline level and reached the lowest value 80 min after TSST (*p* > .05) (Fig. 1a). For non-responders, cortisol levels followed a decreasing trend over time. Overall, BP and HR peaked at the speech stage of the TSST (*p* < .001). While BP gradually declined and reached its lowest level during recovery without returning to baseline (*p* < .001), HR returned to baseline levels in recovery phase (*p*s > 0.05) (Figs. 1b and 1c). The four generalized additive models explained 78.2–85.4 % of the variance of physiological responses during the experiment.

**Table 1:** Demographic characteristics of the sample.

Demographic variable	Sample
	*N* = 248
Age	
18–24	16 (6.5 %)
25–34	187 (75.4 %)
35–45	45 (18.1 %)
Mean age (SD)	30.63 (4.61)
Gender	
Male	109 (44.0 %)
Female	139 (56.0 %)
Monthly household income (HK$)	
0–14,999	6 (2.4 %)
15,000–29,999	40 (16.1 %)
30,000–44,999	65 (26.2 %)
45,000–59,999	45 (18.1 %)
60,000–100,000	70 (28.2 %)
100,000+	22 (8.9 %)
Marital status	
Unmarried	176 (71 %)
Married/Cohabited	72 (29 %)
Employment status	
Full-time	184 (74.2 %)
Non-full-time/Students	64 (25.8 %)
Educational attainment	
Below Bachelor’s degree	40 (16.1 %)
Bachelor’s degree or above	208 (83.9 %)
Medical conditions	
Yes	38 (15.3 %)
No	210 (84.7 %)

**Table 2:** Descriptive statistics of the three physiological measures for all timepoints.

Physiological measures	*N* = 248				
	*M*	*SD*	Median	Range	
				Min	Max
Salivatory Cortisol (ng/ml)					
Baseline	1.38	1.04	1.06	0.10	5.84
Post TSST	2.49	2.00	2.05	0.25	15.10
+20mins Post TSST	3.16	2.77	2.29	0.24	15.85
+40mins Post TSST	2.03	1.62	1.63	0.23	11.03
+60mins Post TSST	1.46	1.00	1.23	0.12	5.78
+80mins Post TSST	1.16	0.75	1.03	0.09	4.86
Peak Cortisol	3.61	2.78	2.75	0.40	15.85
AUCi	63.35	106.34	59.29	–239.84	610.35
Blood Pressure (mm Hg)					
Baseline	81.05	11.10	79.67	56.33	120.33
TSST-Preparation	92.56	12.17	91.50	67.33	130.67
TSST-Speech	107.39	13.02	107.17	54.00	148.67
TSST-Calculation	103.35	13.33	101.83	66.67	147.33
Recovery	82.44	11.10	81.33	57.33	128.33
Peak Blood Pressure	110.65	11.95	109.17	84.33	148.67
AUCi	644.66	105.42	632.59	432.3	1046.21
Heart Rate (BPM)					
Baseline	67.75	10.17	68.00	31.00	97.00
TSST-Preparation	78.72	13.82	78.00	49.00	133.00
TSST-Speech	92.62	18.52	90.50	49.00	148.00
TSST-Calculation	85.99	15.51	85.00	46.00	132.00
Recovery	67.41	9.85	67.00	45.00	101.00
Peak Heart Rate	95.09	17.56	93.00	49.00	148.00
AUCi	450.02	262.18	407.46	–176.22	1420.84

*Note.* BPM = beats per minute. Cortisol data of two participants was missing. AUCi = area under the curve with respect to increase.

**Table 3:** Generalized Additive Models with Salivary Cortisol, Blood Pressure, and Heart Rate as the Respective Outcome Variables, Sampling Time as the Predictor, and Time and Participant as Random Effects.

	Cortisol (responders)				Cortisol (non-responders)				Blood Pressure				Heart Rate			
	Est.	*SE*	*T*	*p*	Est.	*SE*	*T*	*p*	Est.	*SE*	*T*	*p*	Est.	*SE*	*T*	*p*
**Parametric Terms**																
Intercept	0.409	0.042	9.726	< 0.001	0.142	0.089	1.597	.112	4.524	0.007	669.9	< 0.001	4.344	0.009	469	< 0.001
	*edf*	*rdf*	*F*	*p*	*edf*	*rdf*	*F*	*p*	*edf*	*rdf*	*F*	*p*	*edf*	*rdf*	*F*	*p*
**Smooth Term**																
Time	6.304	6.911	75.280	< 0.001	3.134	3.812	16.675	< 0.001	6.023	6.743	18.329	< 0.001	5.913	6.534	74.437	< 0.001
Time, Participant	506.090	1210.00	4.968	< 0.001	82.369	262.00	7.574	< 0.001	239.342	1238.00	1.831	< 0.001	331.914	1238.00	2.313	< 0.001
R^2^_adjusted_	.782				.854				.793				.791			
Deviance Explained	.917				.915				.839				.856			

Note. *edf* = effective degrees of freedom; *rdf* = reference degrees of freedom.

### Physiological functioning scores

The KMO indexes (0.56–0.60), the determinant values (0.87–0.95), and the Bartlett’s tests (χ^2^(3)s =13.71–34.00, *p*s < 0.005) suggested suitability for conducting PCA. Eigenvalues-greater-than-one criterion determined that the physiological functioning of peak reactivity and recovery can be represented by one component, respectively. Each component was of strong positive associations with the raw scores of each dimension (*r*s = 0.60–0.72). For each PCA, the ratio of subjects to variables (∼ 0.83), the number of component (k = 1), and the large component loadings (∼ 0.60) suggested reliable solution regard to the current sample size (Osborne & Costello, 2004). The reactivity and recovery component scores explained 48 % and 43 % variance of the data, respectively. Given that the variance explained by a single principal component in each analysis was close to 50 %, we believe that the solution effectively reflected individual differences in physiological functioning related to reactivity and recovery. The reactivity scores were positively correlated with the recovery scores (*r* = 0.45, *p* < .001), suggesting that high levels of stress reactivity may be associated with slower recovery. The raw PCA scores were standardized to T-score before being used to calculate the resilience index. Table 4 summarizes the PCA results and descriptive statistics of the PCA scores and resilience index.

**Table 4:** PCA results and descriptive statistics of the PCA scores and resilience index.

	PCA Reactivity	PCA Recovery	Resilience Index
KMO index	.60	.56	-
Determinant values	.87	.95	-
Bartlett’s tests	*χ*^2^ (3) = 34.00, *p* < .00001	*χ*^2^ (3) = 13.71, *p* = .00332	-
Component loadings			
Cortisol	.72†	.67‡	-
Blood pressure	.66‡	.60‡	-
Heart rate	.70†	.68	-
Variance	48 %	43 %	-
Intercorrelations			
PCA Reactivity	-	.45 (*p* < .001)	-
Mean	50.00	50.00	–49.70
Standard deviation	10.00	10.00	8.66
Median	50.93	49.67	–49.40
Min	20.83	25.80	–73.58
Max	74.62	79.46	–28.30
Skewness	–0.31	0.32	–0.24
Kurtosis	0.15	–0.16	–0.27
Shapiro-Wilk *p*	.062	.081	.149

*Note*. KMO = Kaiser-Meyer-Olkin. PCA = Principal Component Analysis. PCA scores were standardized to T-score. Resilience index = $-\sqrt{\text{PCA}\;\text{Reactivity}\;\times \;\text{PCA}\;\text{Recovery}}$.

† Inverse hyperbolic sine transformation was applied to the raw scores before doing the PCA, median of the absolute value was used as the scaling constant.

‡ Log transformation was applied to the raw scores before doing the PCA.

### Validity of resilience index

A significant inverse association was observed between the resilience index and psychological distress (*β* = −.115, 95 % Confidence Interval [−.221, −.008], *p* = .035, η²_p_ = .019), controlling for exposures to daily stressors and major life events, and demographic factors. The interaction effect between daily stressors and the resilience index was not significant.

### Predicting resilient acute stress responses

**Fig. 2:**
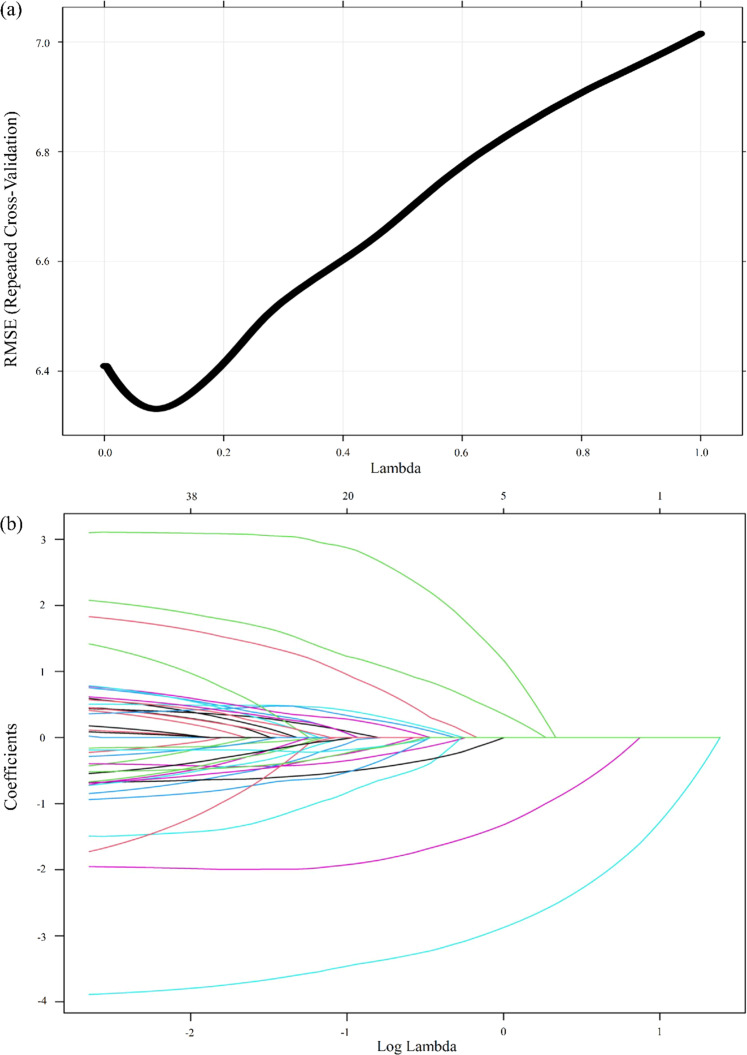
(a) Plot of root mean squared error (RMSE) versus lambda. (b) Plot of least absolute shrinkage and selection operator coefficient profiles against the log lambda sequence.

**Fig. 3:**
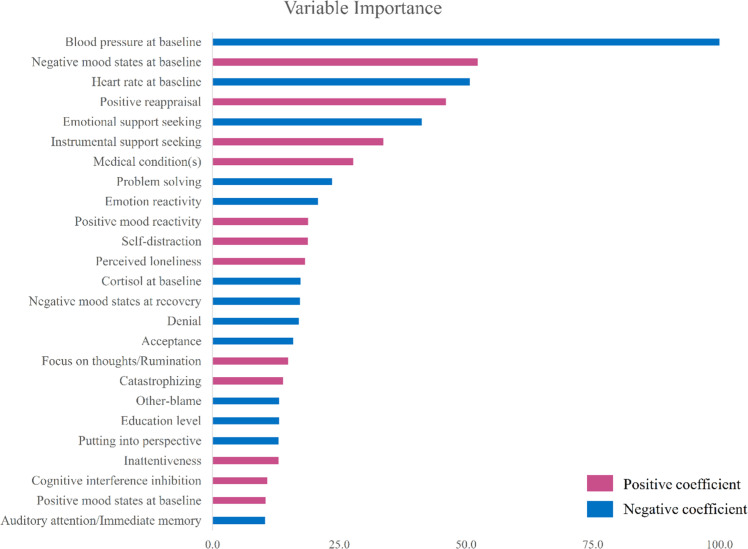
Relative importance of variables in the LASSO regression model predicting resilient acute stress responses. Variable importance <10 were omitted.

Figs. 2a and 2b show the plot of RMSE versus lambda and the LASSO coefficient profiles plot against the log lambda sequence, respectively. The RMSE followed a U-shaped pattern, initially decreasing until it reached its lowest point at a lambda value of 0.087, after which it started to rise with higher lambda values. The magnitude of coefficients also shrank as lambda increased. The final LASSO model with the lowest RMSE identified 25 predictors with variable importance above 10 (*M*_RMSE_ = 6.33, *SD*_RMSE_ = 0.61, *M*_MAE_ = 4.99, *SD*_MAE_ = 0.51, *M_R_*^2^ = 0.43, S*D_R_*^2^ = 0.10). The most important predictors included baseline physiological activity, coping and emotion regulation strategies (e.g., positive reappraisal, instrumental support seeking, emotional support seeking, self-distraction), mood states at baseline, maintenance of positive mood during stress, negative mood at recovery, trait emotion reactivity, chronic stress, cognitive functioning (cognitive interference inhibition and inattentiveness), and demographic factors (minor medical condition(s) (dermatitis and asthma) and education level) (Table 5). Fig. 3 shows the relative importance of variables in the prediction model. Predictors with variable importance < 10 were omitted. The LASSO model refitted on the original participants demonstrated comparable model performance (RMSE = 6.54, MAE = 5.08) and *R*^2^ of 0.43.

**Table 5:** Coefficients and variable importance for all nonzero predictors in least absolute shrinkage and selection operator regression.

Predictors	Coefficient	Variable Importance
Physiological functioning at baseline		
Cortisol	–0.671	17.4
Blood pressure	–3.865	100.0
Heart rate	–1.961	50.7
Mood states		
Positive mood at baseline	0.405	10.5
Negative mood at baseline	2.023	52.3
Positive mood at recovery	0.375	9.7
Negative mood at recovery	–0.668	17.3
Positive mood reactivity	0.729	18.9
Negative mood reactivity	0.140	3.6
Stress exposure and perceived stress		
Major life events over past 5−year	0.248	6.4
Daily stressor(s) over past month	0.089	2.3
Perceived stress over past 3−month	–0.368	9.5
Affective styles		
Trait positive affect		
Trait negative affect		
Emotion reactivity	–0.805	20.8
Protective and risk factors		
Trait resilience		
General self−efficacy		
Perceived loneliness	0.705	18.2
Coping and cognitive emotion regulation strategies		
Self−distraction	0.727	18.8
Instrumental support seeking	1.302	33.7
Positive reappraisal	1.779	46.0
Acceptance	–0.616	15.9
Behavioral disengagement	–0.260	6.7
Catastrophizing	0.540	14.0
Denial	–0.658	17.0
Emotional support seeking	–1.594	41.2
Humor	–0.189	4.9
Other−blame	–0.509	13.2
Positive refocusing		
Problem solving	–0.913	23.6
Putting into perspective	–0.504	13.0
Religion		
Focus on thoughts/Rumination	0.578	14.9
Self−blame		
Substance use	0.066	1.7
Venting		
Cognitive functioning		
Immediate memory	–0.402	10.4
Working memory		
Cognitive interference inhibition	0.419	10.8
Cognitive flexibility	–0.187	4.8
Inattentiveness	0.502	13.0
Demographic characteristics		
Gender^a^	–0.077	2.0
Age		
Education level^b^	–0.507	13.1
Marital status^c^	–0.295	7.6
Employment status^d^	0.178	4.6
Monthly household Income^e^	0.178	4.6
Medical condition(s)^f^	1.074	27.8

*Note.*^a^ Female = 0, Male = 1. ^b^ Non−degree = 0, Degree = 1. ^c^ Non−married = 0, Married = 1.

d Non−full time = 0, Full−time = 1. ^e^ 0 = Below HK$45,000, 1 = HK$45,000 or above. ^f^ No = 0, Yes = 1.

## Discussion

This study constructed and tested a resilience index based on multiple physiological responses in the Trier Social Stress Test (TSST). We adopted a machine learning approach to identify important predictors of resilient acute stress responses which were characterized by low reactivity and/or high recovery in response to acute stress. Predictors were identified from physiological (baseline activity), emotional (positive affective style and emotional reactivity), behavioral (coping and emotion regulation strategies), and cognitive (executive functions) domains, together with perceived stress as well as demographic factors (education level and minor medical conditions), suggesting that factors from different domains could relate to the resilient responses to acute stress.

A resilience index integrating multiple physiological responses to acute stress was constructed and found to reflect better adaptation to daily stressors. Previous studies investigating the association between different stressors and psychological distress in the context of acute stress reactivity and recovery have typically relied on single physiological indicators or used multiple modalities in isolation (e.g., Beech et al., 2023; Quinn et al., 2018; Steeger et al., 2017). Because responses to a single episode of acute stress might not fully capture an individual’s resilience/vulnerability to stress, measures of the levels of cortisol, blood pressure, and heart rate were used as multiple physiological indicators of resilience in order to enhance their validity (Nebe et al., 2023). Although resilience has been operationalized and quantified with reference to an individual's trajectory of mental health outcome after encountering significant traumatic events (e.g., Bonanno et al., 2023) or individual's mental health outcome after adjusting for their cumulative stress load (e.g., Kalisch et al., 2021), relatively less is known about its manifestation during acute stress. This study contributed to the existing evidence by demonstrating that resilient responses to acute stress were inversely associated with psychological distress. Recent technological advancements on wearable devices have facilitated real-time stress detection and continuous monitoring of multiple biological responses to stress (Can et al., 2019; Tang et al., 2021). Our findings suggested that tracking reactivity of and recovery from acute stress in daily life is a feasible direction for the timely identification of resilience and vulnerability to mental health disorders. Measuring physiological responses to various stressors in real-life contexts would also enhance our understanding of an individual's allostatic load beyond what is observed in laboratory settings. Ecological momentary assessment and wearable devices could be leveraged to investigate the causal relationship between resilient responses to acute stress and lower levels of psychological distress in respond to daily stressors.

The most important predictor was physiological functioning at baseline/before the TSST. In particular, lower cardiovascular and cortisol activity levels were associated with resilient responses. Lower resting HR and BP may reflect habitual healthy lifestyle such as regular physical activity (Childs & de Wit, 2014; Igarashi & Nogami, 2020; Reimers et al., 2018), which are beneficial to foster better psychological adaptation under both acute and chronic stressful events (e.g., Childs & de Wit, 2014; Liang et al., 2025; Salmon, 2001). In addition, our findings suggested appropriate timing and modifiable factors that could be targeted for stress adaptation. A wearable device with the capability to perform real-time detection and intervention could be developed. For example, recent synthesized studies have demonstrated that slow-paced breathing could demonstrate reliable effects in inducing short-term improvements in cardiovascular functions (Laborde et al., 2022; Shao et al., 2024). Regular training of slow-paced breathing during and after stressful conditions may thus be a simple and feasible intervention of facilitating resilient physiological responses to stress.

Consistent with previous evidence on the role of positive affect in stress adaptation and resilience (Ong et al., 2006; van Steenbergen et al., 2021), individuals with higher maintenance of positive mood during TSST, higher positive mood states before acute stress and at recovery, lower negative mood states at recovery, together with lower (negative) emotional reactivity and lower perceived stress were more likely to be associated with resilient stress responses. Previous experimental studies have showed that positive emotions could dampen cortisol reactivity and facilitate cardiovascular recovery to negative events (Fredrickson & Levenson, 1998; Speer & Delgado, 2017). Cardiovascular recovery to stress induced in laboratory settings could be hampered by negative affect (Radstaak et al., 2011). A growing body of studies has demonstrated the relationships between affective responses to daily stressors and health outcomes (Ong & Leger, 2022). Greater increases in negative affect and decreases in positive affect in response to school stressors have been found to predict higher levels of internalizing symptoms over a 3-year period in children aged 8 to 13 years (Bai et al., 2020). Similarly, lower reactivity of negative affect and greater maintenance of positive affect to daily racial discrimination predicted lower depressive symptoms among a sample of African Americans (Ong et al., 2018). Resilient physiological responses conceptualized as low reactivity and/or high recovery in stress responses could have a mediatory role in the associations between positive and negative affect and mental health (Kalisch et al., 2015). Sustaining positive affect and dampening the reactivity of negative affect in the face of adversity could benefit more adaptive physiological stress responses, which, in turn, will be associated with the maintenance of mental health.

Notably, the positive impact of affect regulation on resilient stress responses could be preferably achieved through behavioral approaches, such as engaging in distraction activities. This is evidenced by the positive association between the resilience index and habitual use of self-distraction strategy. The effectiveness of positive refocusing may depend on relevant executive functions such as cognitive flexibility and interference inhibition (Dickson & Ciesla, 2018). Effective refocusing requires individuals to shift their thoughts from the ongoing stressor to positive experience while inhibiting the interference of negative thoughts and affect that may arise from stressors (Joormann, 2010), thereby facilitating the recovery of physiological functioning during and after stress (Brosschot, 2010; Ottaviani et al., 2016; Verkuil et al., 2010). Consistent with this notion, our results demonstrated that cognitive interference inhibition were positively associated with resilient stress responses. In contrast, distraction activities may demand less cognitive effort but full engagement, which can improve positive affect and foster sense of mastery (Brans et al., 2013; Waugh et al., 2021). In line with this, distractibility, as indicated by the inattentiveness measure of the continuous performance test and worse auditory attention, was positively associated with the resilience index. In addition, a positive association was observed between focus on thoughts/rumination strategy and the resilience index. Although rumination typically involves dwelling on the reasons behind one’s feelings, the focus on thoughts/rumination items in the cognitive emotion regulation questionnaires in this study primarily reflect a level of introspection about emotions. This introspection may serve as a precursor to other emotion-focused strategies, such as emotional acceptance and emotional expression (Blanke et al., 2020; Roth et al., 2019).

Leveraging a machine learning approach, this study also revealed nuanced associations between habitual coping and emotion regulation strategies and physiological responses to TSST. Positive reappraisal, identified as a key resilient factor and mechanism in previous research (Troy et al., 2023), was positively associated with the resilient stress responses. Furthermore, seeking instrumental support as an active coping process can enhance an individual's sense of worth, self-esteem, and belonging, which may reduce physiological arousal and emotional distress (Thoits, 2011). This support alleviates the burdens of challenging situations, lowering perceived threats and the physiological and emotional impact of stressful events (Thoits, 2011). Conversely, an inverse association was observed between habitual emotional support seeking and resilient responses to the TSST. While emotional support generally acts as a protective factor against stress (Mathieu et al., 2019), the participants were unable to seek timely emotional support during the laboratory experiment after the TSST, prolonging their stress responses relative to the time when they have access to support. In contrast, participants reporting higher perceived loneliness were more likely to exhibit resilient stress responses in this circumstance. In the same vein, a previous study showed that participants who received low emotional support after a stress induction task had slower cortisol recovery if they had a high preference for emotional support (Priem & Solomon, 2015). Similarly, an inverse relationship was observed between problem-solving strategies and the resilience index. Individuals with higher trait planning and active coping may show adequate stress responses that facilitate subsequent proactive coping when situations are controllable (Sladek et al., 2016). However, in the context of the TSST where the acute stressor is largely uncontrollable, these strategies may have little effect, potentially prolonging heightened stress reactivity.

Our findings additionally uncovered the relationships between different mental anticipatory processes and acute stress responses. The positive associations of negative mood states before acute stress and catastrophizing (i.e., overestimating the severity of the event) with low reactivity and/or fast recovery during the TSST suggested that considering a worst-case scenario may prepare themselves mentally for potential challenges, which may enhance predictability and reduce perceived threat once the situation unfolds congruently or less seriously over time (Fu et al., 2024; Wang et al., 2021). However, it is important to note that while a negative outlook can have a protective effect for coping with acute stress, enduring catastrophizing could be related to psychological distress and hinder effective coping (Li et al., 2024). On the other hand, the inverse association between putting-into-perspective and resilience index indicated that comforting oneself by viewing the stressor as less significant may backfire if the situation worsens and relate to increased feelings of uncontrollability and heightened stress responses (Fu et al., 2024; Wang et al., 2021). Similarly, mindsets of helplessness, avoidance, and an external locus of control - manifested as acceptance of the situation, denial, and other-blaming - can hamper proactive coping (e.g., positive reappraisal), thereby exaggerating and prolonging stress reactivity (Perez et al., 2023). Our findings suggested that the interplay between personal regulatory disposition and the nature of adversity significantly influences acute stress responses. Higher perceived stress, reflecting a long-term imbalance between situational demands and perceived coping capacity, was associated with higher levels of stress responses. Importantly, no single strategy is universally effective for all stressors. Therefore, a diverse repertoire of regulatory strategies is needed for flexible application according to the specific characteristics of the stressful context and flexible adaption of responses based on feedback from previous coping efforts (Bonanno & Burton, 2013; Bonanno et al., 2023).

This study further showed the important interactions between life experiences and the nature of adversity in shaping resilience (Troy et al., 2023). Lower educational attainment was associated with a higher resilience index, possibly because individuals with less education tend to use fewer problem-focused coping strategies (Cheng, 2001), which was associated with higher levels of resilience index in the context of TSST. Current and/or past medical condition(s), most commonly dermatitis and asthma, could reflect increased coping capacity consequential to daily hassles such as relatvely minor and manageable medical issues (Rutter, 2012; Seery, 2011). However, cautions are warranted in interpreting and generalizing the findings to persons with major physical illnesses especially cardiovascular disease, diabetes, and cancer, because such people are at a higher risk of developing mental health problems (Doherty & Gaughran, 2014). More in-depth investigation is needed to elucidate the associations between different demographic and medical characteristics and physiological responses to different stressor(s).

## Limitations

Several limitations should be acknowledged in this study. First, our research was conducted among young and middle-aged healthy adults without any history(ies) of major psychiatric and physical condition(s), thus the findings cannot be readily extrapolated to older adults or people with psychiatric condition(s). Second, although the sample size was comparable to that of previous studies (Kotlicka-Antczak et al., 2019; Koutsouleris et al., 2018), it was relatively small, which could introduce bias and limit the generalizability of the results. To enhance the reliability of model performance estimates, we utilized random resampling for data augmentation and conducted a 10-fold cross-validation with 50 repetitions. These procedures helped validate the results internally and reduce the risk of overfitting in the prediction model. Third, the recovery of heart rate and blood pressure was assessed based on their levels after a specific duration of time, limiting its sensitivity to capture individual differences in cardiovascular recovery. Future studies could consider employing continuous measurements to determine the time taken for participants to return to baseline.

## Conclusion

Notwithstanding the limitations, our findings contribute to a deeper understanding of resilient physiological responses to acute stress. Low reactivity and/or rapid recovery following acute stress could be adaptive in the face of acute stressful conditions in our everyday life. They could represent short-term physiological consequences through which different resilient factors operate to contribute to the long-term maintenance of mental health. The current findings could point to feasible directions for developing interventions for enhancing individual resilience and promoting mental well-being in everyday life. Informed by real-time stress detection technique, cognitive behavioral strategies such as slow-paced breathing (Laborde et al., 2022; Shao et al., 2024) coupled with positive emotions and a realistic pessimistic outlook could be timely used to facilitate resilient physiological responses during and after stressful conditions.

## Funding source

This work was supported by the Central Reserve Allocation Committee, The Education of University of Hong Kong, Hong Kong SAR, China [WKH, number: 04A39]. The funding source had no role in any process of our study.

## Declaration of competing interest

The authors declare that they have no conflict of interest.

## Appendix Supplementary materials

Image, application 1
