# Supplementary material for: Predicting acute stress recovery: A resilience index of physiological responses to Trier Social Stress Test
Source: Int J Clin Health Psychol. 2025 Dec 18;26(1):100652. doi: 10.1016/j.ijchp.2025.100652 (PMC12775958; doi:10.1016/j.ijchp.2025.100652)

**Supplementary Material.**

**Psychometric measures and neuropsychological tests**

Daily stressors. The Daily Hassle Scale (DHS) is consisted of 63 items that assess daily stressors over the past month (Holm & Holroyd, 1992). Participants indicated the frequency of occurrence for each item. This scale comprised covert (e.g., inner concerns) and overt hassles (e.g., environmental hassles). Total scores of the 63 items were computed to indicate the overall exposures to daily stressors in the last month.

Major life events. The Life Stress Index (LSI) assesses the experience of 17 major life events (e.g., natural disaster, traffic accident), generated from the Life Stress Assessment (Nan et al., 2012) and the Life Events Checklist for DSM-5 (Weathers et al., 2013). Participants indicated whether they had personally experienced the event or witnessed the event happening to others, in the past five years. For each event, the participant was also asked to provide details about the event’s frequency and duration, as well as a brief description of the event. For witnessing events, only those happening to the participant’s close friend or family member counted. Number of events was summed with 1 granted for every personal experience of a life event, and 0.5 for every witnessing of the event. Higher scores indicated greater exposures to different major life stressful events. Further details about the life stress assessment can be referred to a previous publication using the same instrument (Shao et al., 2023). A binary variable was generated. Participants who experienced 1 or fewer major life events were coded as 1, while those who experienced more than 1 major life event were coded as 0.

Psychological distress. The 28-item General Health Questionnaire was used to assess participants’ psychological distress (Goldberg & Hillier, 1979). This instrument comprises four subscales: somatic symptoms, anxiety/insomnia, social dysfunction, and severe depression. Participants rated their experience over the past three month on a 4-point Likert scale, ranging from 0 (better than usual/not at all) to 3 (much worse than usual/much more than usual). The GHQ has shown satisfactory psychometric properties among Chinese population (Chan, 2002; Shek, 1989). The total score was calculated, with higher scores indicating greater levels of psychological distress. In the current administration, Cronbach’s alpha was 0.89, demonstrating good internal consistency.

Emotion States. The Profile of Mood States (POMS) was used to assess transient mood states at baseline, after the TSST, and during recovery (Grove & Prapavessis, 1992). It consists of 40 items that were rated on a 5-point Likert scale ranging from 0 (not at all) to 4 (extremely). The POMS measures five negative emotion states (tension, anger, fatigue, confusion and depression) and two positive emotion states (vigour and esteem). Average scores for positive and negative moods were computed for baseline, post-TSST, and during recovery, with higher scores indicating higher levels of each emotion state. The POMS demonstrated excellent internal consistency in the current sample, with Cronbach's alpha coefficients ranging from 0.89 to 0.96. Notably, mood reactivity was determined as the difference between baseline and post-TSST scores.

Perceived stress. The Perceived Stress Scale (PSS; Cohen et al., 1983) is a 10-item measure that was developed to assess the extent to which individuals perceive situations as stressful. The Chinese version of the PSS, which has demonstrated satisfactory psychometric properties among Hong Kong Chinese (Leung et al., 2010), was administered in the current study. Participants were asked to report how frequently they experienced certain feelings and thoughts over the past month, using a 5-point Likert-type scale (0 = never, 4 = very often). Four positively worded items were reverse-scored before being summed. Higher total scores indicated higher levels of perceived stress. Cronbach’s alpha was 0.86.

Coping and Cognitive Emotion Regulation Strategies. To assess the frequency of various coping and emotion regulation strategies in response to stressful or negative experiences, we utilized the Brief-COPE (Carver et al., 1989) and the short form of the Cognitive Emotion Regulation Questionnaire (CERQ) (Garnefski & Kraaij, 2006). The Brief-COPE includes 14 coping strategies, while the CERQ short form comprises 9 cognitive emotion regulation strategies, some of which overlap. For example, both the positive reframing subscale of the COPE and the positive reappraisal subscale of the CERQ focus on reinterpreting problems positively, and the acceptance and self-blame subscales reflect similar approaches to dealing with challenges. Consequently, we integrated the overlapping strategies, resulting in 18 distinct coping and emotion regulation strategies: instrumental support seeking (Cronbach’s alpha (α) = 0.83), positive reappraisal (α = 0.79), self-distraction (α = 0.58), acceptance (α = 0.76), behavioral disengagement (α = 0.76), catastrophizing (α = 0.74), denial (α = 0.65), emotional support seeking (α = 0.84), humor (α = 0.76), other-blame (α = 0.71), positive refocusing (α = 0.64), problem solving (α = 0.85), putting into perspective (α = 0.65), religion (α = 0.74), focus on thoughts/rumination (α = 0.79), self-blame (α = 0.75), substance use (α = 0.95), and venting (α = 0.65). Subscale scores were obtained by averaging the relevant items, with higher scores indicating greater use of each strategy. The current administration exhibited acceptable to excellent internal consistency.

Trait Affect. The Chinese Affect Scale (CAS) (Hamid & Cheng, 1996) is a 20-item measure that assesses positive and negative affect. Participants were asked to rate how they feel each affect in general on a 5-point Likert scale ranging from 1 (very slightly or not at all) to 5 (extremely). Two subscale scores were calculated by summing the relevant items of positive affect and negative affect, respectively, with higher scores indicating higher levels of that type of affect. The CAS showed good internal consistency, with Cronbach's alpha coefficients of 0.91 for positive affect and 0.89 for negative affect.

Emotion Reactivity. The Emotion Reactivity Scale (ERS) (Nock et al., 2008) was used to assess one’s emotional sensitivity, intensity, and persistence in daily life. Participants indicated the extent to which each item describe themselves by rating them on a 5-point Likert scale ranging from 0 (not at all like me) to 4 (completely like me). Total scores were calculated for all the items, with higher scores indicating greater disposition of emotional reactivity. The ERS demonstrated excellent internal consistency, with Cronbach's alpha coefficient of 0.95.

Trait resilience. The Chinese version of the Connor-Davidson Resilience Scale (CD-RISC) (Connor & Davidson, 2003) was used to assess participant’s trait resilience (Yu & Zhang, 2007). It comprises 10 items that participants rated with reference to their experience in the past month on a 5-point Likert scale ranging from 0 (not true at all) to 4 (true nearly all the time). A higher total score indicated greater trait resilience. The Chinese version of the CD-RISC has shown satisfactory psychometric properties among Chinese population (Ni et al., 2016). Cronbach’s alpha was 0.91 in the current administration.

Self-efficacy. The 8-item New General Self-Efficacy (NGSE) scale was used to assess one’s general self-efficacy (Chen et al., 2001). Participants rated the items on a 5-point Likert scale ranging from 1 (strongly disagree) to 5 (strongly agree). A total score was calculated, with higher scores indicating greater general self-efficacy. The NGSE demonstrated good internal consistency (Cronbach's alpha = 0.92).

Perceived Loneliness. The 6-item UCLA-Loneliness Scale (Neto et al., 2014) is a brief measure of loneliness. Participants were asked to indicate the frequency with which they experience each item on a 4-point Likert scale ranging from 1 (never) to 4 (always). A total score was calculated, with higher scores indicating greater perceived loneliness. The scale demonstrated good internal consistency in the current sample (Cronbach's alpha = 0.87).

Auditory attention/Immediate Memory. The Digit Span Forward subtest of the Wechsler Adult Intelligence Scale-Fourth Edition (Hong Kong) [WAIS-IV (HK)] (Wechsler, 2008) was used to assess auditory attention and immediate memory. In this task, the administrator verbally presented the participant with a series of numbers, and the participant was instructed to repeat the digits in the exact same order. The length of the number sequences gradually increased throughout the assessment, with two trials presented at each level. The test continued until the participant failed to correctly recall both number sequences within a given length. The total number of successfully repeated trials was recorded, with higher scores indicating better attention and immediate memory.

Working Memory. The Digit Span Backward, Digit Span Sequencing, and Arithmetic subtest of the WAIS-IV (HK) (Wechsler, 2008) were administered to evaluate working memory. In the two Digit Span tasks, participants were instructed to repeat verbally presented number sequences in either reverse order (Backward) or ascending order (Sequencing). The total number of successfully repeated trials was recorded for each subtest. The Arithmetic subtest required participants to mentally compute answers to a series of increasingly complex word problems, without the use of paper and pencil. Participants had to accurately solve the problems within a specified time limit. Scores were based on the number of correct responses. A composite score was calculated by summing the raw scores across the three subtests, with higher scores representing better working memory.

Cognitive Flexibility. The Trail Making Test (TMT) (Lee & Wang, 2010) was used to assess cognitive flexibility. The test consists of two parts: Part A and Part B. In Part A, the participant was asked to connect a series of numbered circles in sequential order. In Part B, the participant had to alternatively connect numbered and lettered circles in sequential order. The time taken to complete each part of the test was recorded. A performance score was calculated by subtracting the completed time of Part A from the completed time of Part B and was divided by completed time of Part A, and then multiplied by –1. A higher score indicates better performance.

Inattentiveness. The Conner Continuous Performance Test-3 (CPT-3) (Conners, 2014) was used to assess inattentiveness. This computerized task presented participants with a series of letters on a computer screen. Participants were instructed to refrain from pressing the space button whenever non-target letters (i.e., the letter "X") appeared, but to press the space button when target letters (i.e., all other letters) were displayed. The letters were presented at a rapid pace, and the participant had to maintain focus and respond accurately throughout the approximately 14-minute test duration. The detectability index (d’) generated by the CPT-3 was used to indicate participants’ inattentiveness. It is reverse-scored, meaning that higher scores represent poorer attention.

Cognitive Inhibition. The Chinese version (Lee & Wang, 2010) of the Stroop Colour-Word Test (SCWT) Victoria Version (Regard, 1981) was employed to assess inhibition of cognitive interference. The SCWT consists of three cards printed with dots, non-color common words, and color words, respectively. Each card contains 24 items arranged in a 4 × 6 matrix. Yellow, blue, green, and red were used for the stimuli, with each color appearing once per row in a pseudorandomized order. The stimulus card was presented one at a time, and participants were instructed to rapidly name the ink color of each stimulus, while disregarding the verbal content. The time (in seconds) for completing each stimulus card was recorded. An interference score was calculated using the formula ([time (color-words) – time (dot)]*(–1)), with higher scores indicating better inhibition of cognitive interference.

**References**

Carver, C. S. (1997). You want to measure coping but your protocol's too long: Consider the brief cope. *International Journal of Behavioral Medicine, 4*, 92–100. https://doi.org/10.1207/s15327558ijbm0401_6

Chan, D. W. (2002). Stress, self-efficacy, social support, and psychological distress among prospective Chinese teachers in Hong Kong. *Educational Psychology*, *22*(5), 557–569. https://doi.org/10.1080/0144341022000023635

Chen, G., Gully, S. M., & Eden, D. (2001). Validation of a new general self-efficacy scale. *Organizational research methods, 4*, 62–83. https://doi.org/10.1177/10944281014100

Cohen, S., Kamarck, T., & Mermelstein, R. (1983). A global measure of perceived stress. *Journal of Health and Social Behavior, 24*, 385–396. https://doi.org/10.2307/2136404

Conners, C. K. (2014). *Conners continuous performance test 3rd edition manual*. Toronto: Multi-Health Systems.

Connor, K. M., & Davidson, J. R. (2003). Development of a new resilience scale: The Connor‐Davidson Resilience Scale (CD‐RISC). *Depression and Anxiety, 18*, 76–82. https://doi.org/10.1002/da.10113

Garnefski, N., & Kraaij, V. (2006). Cognitive emotion regulation questionnaire–development of a short 18-item version (CERQ-short). *Personality and Individual Differences, 41*, 1045–1053. https://doi.org/10.1016/j.paid.2006.04.010

Goldberg, D. P., & Hillier, V. F. (1979). A scaled version of the General Health Questionnaire. *Psychological Medicine*, *9*(1), 139–145. https://doi.org/10.1017/S0033291700021644

Grove, J. R., & Prapavessis, H. (1992). Preliminary evidence for the reliability and validity of an abbreviated Profile of Mood States (POMS) questionnaire. *International Journal of Sport Psychology, 23*, 93–109.

Hamid, P. N., & Cheng, S. T. (1996). The development and validation of an index of emotional disposition and mood state: The Chinese Affect Scale. *Educational and Psychological Measurement, 56*, 995-1014. https://doi.org/10.1177/0013164496056006006

Holm, J. E., & Holroyd, K. A. (1992). The Daily Hassles Scale (revised): Does it measure stress or symptoms? *Behavioral Assessment, 14*, 465–482.

Lee, T.M.C., Wang, K., 2010. Neuropsychological Measures: Normative Data for Chinese (2nd ed. revised). Laboratory of Neuropsychology, The University of Hong Kong, Hong Kong.

Leung, D. Y., Lam, T. H., & Chan, S. S. (2010). Three versions of Perceived Stress Scale: validation in a sample of Chinese cardiac patients who smoke. *BMC Public Health*, *10*, 513. https://doi.org/10.1186/1471-2458-10-513

Nan, H., Lee, P. H., McDowell, I., Ni, M. Y., Stewart, S. M., & Lam, T. H. (2012). Depressive symptoms in people with chronic physical conditions: prevalence and risk factors in a Hong Kong community sample. *BMC Psychiatry*, *12*, 198. https://doi.org/10.1186/1471-244X-12-198

Neto, F. (2014). Psychometric analysis of the short-form UCLA Loneliness Scale (ULS-6) in older adults. *European Journal of Ageing, 11*, 313–319. https://doi.org/10.1007/s10433-014-0312-1

Nock, M. K., Wedig, M. M., Holmberg, E. B., & Hooley, J. M. (2008). The Emotion Reactivity Scale: development, evaluation, and relation to self-injurious thoughts and behaviors. *Behavior Therapy, 39*, 107–116. https://doi.org/10.1016/j.beth.2007.05.005

Regard, M., 1981. Cognitive rigidity and flexibility: a neuropsychological study [Doctoral dissertation, University of Victoria].

Shao, R., Man, I. S., Yau, S. Y., Li, C., Li, P. Y., Hou, W. K., ... & Lee, T. M. (2023). The interplay of acute cortisol response and trait affectivity in associating with stress resilience. *Nature Mental Health*, *1*(2), 114–123. https://doi.org/10.1038/s44220-023-00016-0

Shek, D. T. (1989). Validity of the Chinese version of the General Health Questionnaire. *Journal of Clinical Psychology*, *45*(6), 890–897. https://doi.org/10.1002/1097-4679(198911)45:6<890::AID-JCLP2270450610>3.0.CO;2-G

Weathers, F. W., Blake, D. D., Schnurr, P. P., Kaloupek, D. G., Marx, B. P., & Keane, T. M. (2013). The Life Events Checklist for DSM-5 (LEC-5). National Center for PTSD. http://www.ptsd.va.gov

Wechsler, D. (2008). Wechsler Adult Intelligence Scale-Fourth Edition. San Antonio, TX: Pearson.

Yu, X., & Zhang, J. (2007). Factor analysis and psychometric evaluation of the Connor-Davidson Resilience Scale (CD-RISC) with Chinese people. *Social Behavior and Personality: an international journal*, *35*, 19–30. https://doi.org/10.2224/sbp.2007.35.1.19

**Figure S1.** Cortisol Levels Over Time for Responders and Non-Responders.


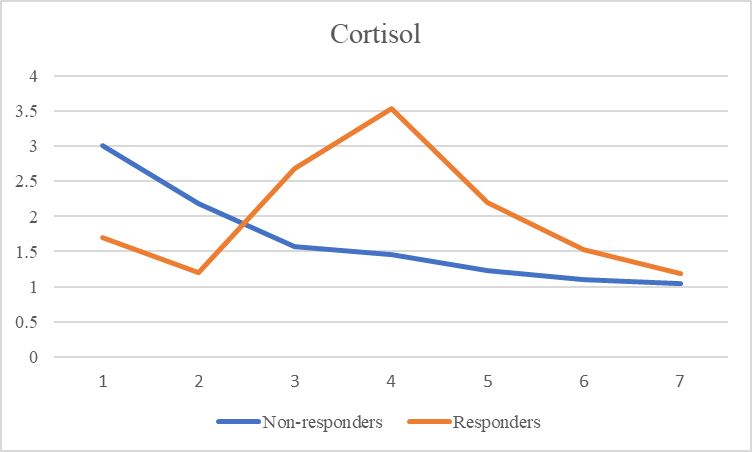


**Figure S2.** Blood Pressure Over Time for Responders and Non-Responders.


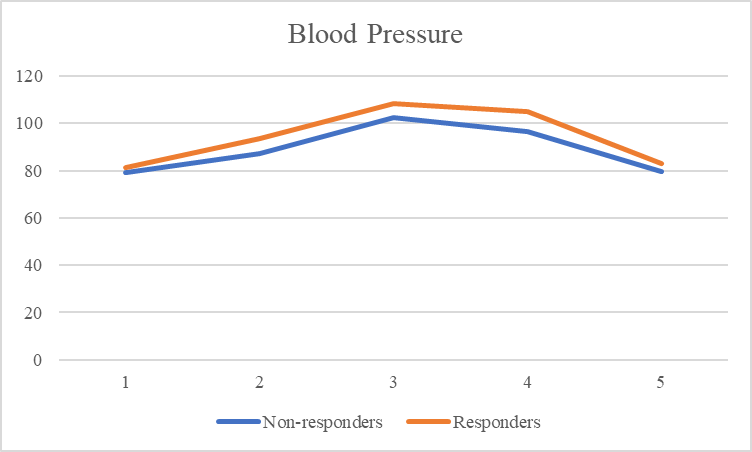


**Figure S3.** Heart Rate Over Time for Responders and Non-Responders.


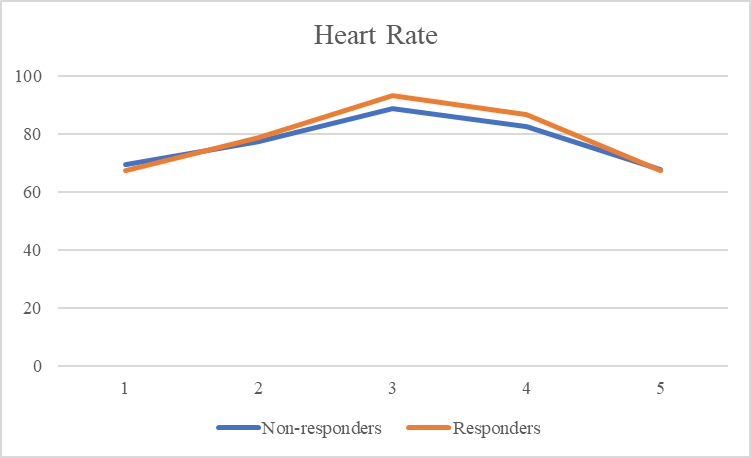

Supplement: Supplementary file 1 [file mmc1.docx]
